# Surgical Pathology and Therapeutics

**Published:** 1895-12

**Authors:** 


SURGICAL PATHOLOGY AND THERAPEUTICS. By John Collins Warren, M. D., Professor of Surgery in Harvard University; Surgeon to the Massachusetts General Hospital. 832 pages; with 132 illustrations and four full page colored plates. Price, cloth, $6; half morocco, $7. W. B. Saunders, publisher, 925 Walnut street, Philadelphia. 1895.

In this work the author has supplied something that is important and eminently practical in the student's curriculum, something that was formerly regarded as being wholly ornamental and unnecessary.

As the author truthfully says in his preface: "No young practitioner can be regarded as thoroughly equipped for surgical work who is not both a good pathologist and an expert bacteriologist. The confidence born of a knowledge of pathology and bacteriology enables him to assume grave responsibilities and to grapple successfully with the most complicated problems." The author realizing the necessity of this knowledge to the successful surgeon, and knowing that a large number of practicing physicians had, at the time of their graduation, but poor opportunities for acquiring it, has here presented, in a readable form, many subject which received but little attention at that time. He has furnished a means of becoming familiar with surgical pathology, together with symptoms and treatment of surgical diseases, and has endeavored to emphasize the value of these lines of study as a firm foundation for good clinical work.

The first chapter covers twenty-six pages and is devoted to bacteriology; the second chapter has thirty-six pages and treats entirely of surgical bacteria. These chapters are of especial interest to the surgeon; in them are discussed subjects which are of prime importance, and they are prepared with so much skill that any medical student can easily compreheud everything contained in them and the dullest will recognize the importance of a knowledge of the subjects under discussion. In these two chapters is contained the real foundation upon which the entire work rests, i. e., it is a book written throughout from the standpoint of a bacteriologist. The book covers the whole range of surgical subjects, beginning with hyperæmia, and including the subjects of simple inflammation, infective inflammation, gangrene, shock, surgical fevers, septicæmia, pyæmia, erysipelas, hospital gangrene, tetanus, hydrophobia, actinomycosis, anthrax, glanders, snake bite, tuberculosis, surgical tuberculosis of joints, tuberculosis of the soft parts, diseases of boue, tumors, carcinoma, sarcoma, benign tumors, etc.

This is one of the most important works issued during the year, and we have no hesitancy in saying that it is by far the best American work on the subject of surgical pathology. H.

